# Comparative Study of the Surgical Excision of Impacted Mandibular Third Molars Using Surgical Burs and an Erbium-Doped Yttrium Aluminum Garnet (Er:YAG) Laser

**DOI:** 10.7759/cureus.49816

**Published:** 2023-12-02

**Authors:** Rameet Sandhu, Harsh Kumar, Rashi Dubey, Divya Vyas, Ajoy K Shahi

**Affiliations:** 1 Department of Oral and Maxillofacial Surgery, Luxmi Bai Institute of Dental Sciences and Hospital, Patiala, IND; 2 Department of Dentistry, Patna Medical College and Hospital, Patna, IND; 3 Department of Pedodontics and Preventive Dentistry, Sharad Pawar Dental College and Research Centre, Wardha, IND; 4 Department of Pedodontics and Preventive Dentistry, Himachal Institute of Dental Sciences, Paonta Sahib, IND; 5 Department of Oral and Maxillofacial Surgery, Dental Institute Rajendra Institute of Medical Sciences, Ranchi, IND

**Keywords:** erbium-doped yttrium aluminium garnet, vas scale, trismus, osteotomy, mouth opening

## Abstract

Background

The use of lasers has increased in the field of dentistry in recent years. However, in the field of oral and maxillofacial surgery, the use of lasers has been largely restricted to soft tissue, and less focus is placed on the use of lasers for hard tissues.

Aim

The present study aimed to comparatively evaluate the efficacy of a erbium-doped yttrium aluminum garnet (Er:YAG) laser for cutting the bone while removing the impacted mandibular third molar with the use of a surgical bur.

Methods

The study assessed 80 subjects undergoing removal of impacted mandibular third molars. The subjects were recruited from the Department of Oral and Maxillofacial Surgery, Luxmi Bai Institute of Dental Sciences and Hospital, Patiala, Punjab, India. They were randomly divided into two groups, each consisting of 40 subjects. Group I used an Er:YAG laser to remove the bone, while Group II used a surgical bur. Assessments and comparisons were made for complications, wound healing, trismus, edema, bleeding, and pain.

Results

The study examined the efficacy of the Er:YAG laser for cutting the bone and extracting the impacted mandibular third molars using a surgical bur in two groups of 80 patients each. When the laser was used in place of a surgical bur, Group I patients experienced less edema, bleeding, and discomfort; nevertheless, the difference was not statistically significant. Time taken by laser for bone cutting was significantly higher compared to the surgical bur. In Group I (laser), trismus existed for a longer time. For mouth opening, the preoperative mouth opening was comparable in the two groups with p = 0.87.

Conclusion

Pain, hemorrhage, and edema were lesser, and the time required for bone cutting was substantially longer in the laser group. Apart from these, laser-assisted intra-oral bone cutting should be preferred because of its less invasive nature, simpler procedure, and simpler osteotomy when compared to traditional surgical burs and also in anxiety-prone patients.

## Introduction

The surgical extraction of an impacted third molar is one of the most frequent procedures performed in the area of oral and maxillofacial surgery. Both soft and hard tissues are involved in the surgical removal of the impacted third molar, which raises worries for maxillofacial surgeons since it may be the cause of a number of postoperative difficulties. After lower impacted teeth are removed surgically, the most frequent consequences that impair the quality of life are trismus, edema, and discomfort [1].

Surgical burs are often used to remove the bone surrounding impacted teeth. However, in recent times, a wide range of disciplines, including surgery, ophthalmology, and dermatology, have greatly expanded their use of lasers. Only the soft tissues have been treated with lasers in the field of oral and maxillofacial surgery. In the realm of oral surgery, carbon dioxide (CO_2_) and ruby lasers were employed for hard tissues [2]. However, because of related problems, such as excessive carbonization, delayed bone healing, and heat injury, their usage was limited [3].

The use of hard tissue lasers has become simpler with the advent of erbium-doped yttrium aluminum garnet (Er:YAG) laser and improvements in the laser field since these lasers produce less heat while cutting hard tissues. Er:YAG laser has been widely employed in in-vitro and in-vivo dental research, and reports of its successful application in clinical trials have been made [4,5].

An in-vivo and animal research on orthopedics have both shown the success of Er:YAG with laser therapy [6]. After being successfully applied in a number of medical domains, Er:YAG was investigated in the craniofacial area. In rats, the Er:YAG laser was shown to be an effective substitute for surgical burs when plating a thin bone when it was used to drill the screws for mandible fixation [7].

The Er:YAG laser has been identified by several studies in the fields of orthopedics and maxillofacial surgery as the most promising laser for accurate and efficient bone removal. Furthermore, Er:YAG lasers are infrared laser systems with brief pulses that may be effectively used to ablate dental hard tissues, including bones. The Er:YAG laser is a solid laser that emits light in the infrared spectrum with a wavelength of 2940 nm, matching and enhancing the water absorption peak. It is an effective laser for cutting dental hard tissues, including the alveolar bone, to a depth of a few millimeters since it is also absorbed by the hydroxyapatite in dental hard tissues [8].

The current study sought to compare the effectiveness of the Er:YAG laser in terms of cutting bones and extracting the impacted mandibular third molar using a surgical bur.

## Materials and methods

The present prospective clinical study aimed to comparatively evaluate the efficacy of the Er:YAG laser for bone cutting while removing impacted mandibular third molars with the use of surgical burs. The study subjects were recruited from the Department of Oral and Maxillofacial Surgery, Luxmi Bai Institute of Dental Sciences and Hospital, Patiala, Punjab, India. We have taken clearance from the concerned Institutional Ethical Committee (approval no. IEC/LBIDSH/2022/104). The study period was 16 months, from February 2022 to June 2023.

After being informed about the treatment plan and potential risks, each participant was required to provide both written and verbal agreements to participate in the trial.

The study's inclusion requirements were people who were willing to participate in the research, had impacted mandibular third molars that needed to be surgically extracted, and had no absolute or relative contraindications to receiving local anaesthetic or having the extraction done. Subjects with radiation allergies, immunosuppressants, anticoagulants, and local or systemic bone disorders, breastfeeding women, and pregnant women were excluded.

A thorough intra-oral clinical examination was conducted after a pre-structured, in-depth history was obtained from each participant. Routine hematologic investigations came next. Intra-oral periapical radiographs (IOPARs) were then used to examine the preoperative evaluation radiographs for the relevant region. The impactions were classified as horizontal, distoangular, or mesioangular impaction types based on the analysis of the radiographs.

The study had 80 participants, 40 of whom were female and 40 were male. Er:YAG laser users made up Group I, and surgical bur users made up Group II. The two groups' separate goals were to use surgical burs and an Er-YAG laser to remove bones from the affected teeth.

The extraction was performed with appropriate local anaesthesia using 2% lignocaine and 1:80,000 adrenaline. Ward's incision was made for extraction, and then the full-thickness mucoperiosteal flap was reflected. Enough care was taken to prevent any harm to the lingual nerve.

For Group I, an Er:YAG laser (Biolase Waterlase iPlus, manufactured by North America Biolase, Inc., CA, USA) was used to cut the bone and tooth, and for Group II, no. 6 surgical bur was used to remove the distal and buccal portions of the bone around the impacted teeth. A large amount of saline irrigation was performed while the surgical bur was being used. All of the surgeons used protective eyewear while utilizing the Er:YAG laser during the surgery. The Er:YAG laser utilized had a wavelength of 2.94 nm and a pulse energy of 700 mJ at 10 Hz. The laser tip was positioned one to two millimeters away from the tooth or bone. There was a lot of irrigation with salt water during the entire process. The elevator was used to deliver the tooth in accordance with the root curvatures. Using a file, any sharp bone borders were designed. After a thorough evaluation, saline was used to irrigate the extraction socket. This was followed by suture closure and approximation.

Every research participant's clinical parameter was noted. The visual analog scale (VAS) was used to measure the participants' pain. The VAS consists of a line, often 10 cm long, with verbal anchors at either end, similar to an NRS, e.g., “no pain” on the far left and “the most intense pain imaginable” on the far right. Mild pain was scored 1-4, moderate pain was scored 5-7, and severe pain was scored 8-10. All participants also had their postoperative complications, wound healing, trismus, swelling, time for bone cutting, and bleeding during tooth extraction recorded. When it came to bleeding, minor bleeding was defined as stopping on its own after 15 minutes of pressure application, moderate bleeding was defined as stopping or slowing down with pressure and then starting up again after pressure removal, and severe bleeding occurred when pressure application failed to stop the bleeding [9].

The lateral canthus of the eye was measured both pre- and postoperatively for quantification of the facial swelling, with the exception of the lingual swelling, and the distance between the corner of the mouth and the center of the tragus of the ear and the angle of the mandible were used to measure the postoperative swelling [10]. The following postoperative problems were evaluated: changed sensation in the lingual or inferior alveolar nerves, hyperesthesia, anaesthesia, paresthesia, and infections, such dry sockets. In terms of healing, it was evaluated based on the following criteria: palpable lymph nodes, pus discharge, change in mucosa colour, chronic swelling, or postoperative discomfort. Every one of these healing characteristics was evaluated as either interrupted or smooth. The assessment of trismus involved measuring the interincisal distance, which is a painless mouth opening.

Statistical analysis

The data gathered were assessed statistically by IBM SPSS Statistics for Windows, version 21 (released 2012; IBM Corp., Armonk, New York, United States). The data were expressed as mean values and standard deviations and percentage and frequency. A p-value of <0.05 signified the level of significance.

## Results

The objective of the current prospective clinical investigation was to compare the effectiveness of the Er:YAG laser in cutting bones and extracting impacted mandibular third molars using a surgical bur. Eighty individuals, forty of whom were male and forty were female, participated in the study. Group I consisted of Er:YAG laser users, while Group II consisted of surgical bur users. The purpose of the two groups was to remove the bone from the impacted teeth using an Er:YAG laser and a surgical bur, respectively.

The mean age of the study participants was 26.4±4.24 and 26.6±4.82 years for Group I and Group II study subjects, respectively. The study had 55% (n = 22) males and 45% (n = 18) females in Group I and 40% (n = 16) males and 60% (n = 24) females in Group II. The distoangular, horizontal, and mesioangular impaction type was seen in 10% (n = 4) subjects, 5% (n = 2) subjects, and 85% (n = 34) study subjects, respectively. In terms of bleeding and pain, no statistical difference was seen in the two groups of study subjects. The time needed for bone cutting was significantly more for laser use compared to the surgical bur use where the time was nearly double.

Group I, which employed an Er:YAG laser, had wound healing in six days, whereas Group II (surgical bur) experienced wound healing in eight days. With p > 0.05, the surgical bur caused a non-significantly longer wound healing period. In Group I, 100% of the patients (n = 40) and, in Group II, 92.5% of the individuals (n = 37) experienced mild bleeding. The results show that postoperative bleeding and swelling are lesser in the laser group. In the surgical bur group, 7.5% (n = 3) had moderate bleeding. Neither group's subjects had severe bleeding. With p > 0.05, the difference in bleeding between the two groups was statistically not significant. A major portion (90%, n = 36) of the respondents in Group I and 80% (n = 32) of the subjects in Group II reported having mild discomfort. Meanwhile, 7.5% (n = 3) of the respondents in Group I and 5% (n = 2) of the subjects in Group II reported having moderate discomfort. In Group 1, 2.5% (n = 1) of the respondents while none of the subjects in Group II reported having severe discomfort. As shown in Table 1, the differences between the two groups were statistically non-significant, with p > 0.05.

**Table 1:** Clinical parameters in the two groups of study subjects % = percentage, n = number of patients

Clinical parameters	Group I (Er:YAG laser)	Group II (surgical bur)	p-value
Wound healing (days)	6	8	>0.05
Intraoperative bone cutting (minutes)	28	17	0.000
Bleeding % (n)			
Mild	100 (40)	92.5 (37)	>0.05
Moderate	0	7.5 (3)	
Pain			
Mild	90 (36)	80 (32)	>0.05
Moderate	7.5 (3)	5 (2)	
Severe	2.5 (1)	0	

Point A was defined as one-fourth the distance from the center of the tragus of the ear and the corner of the mouth, and Point B is one-fourth the distance from the lateral canthus and the angle of the mandible. These measurements were used to analyze the swelling in the two groups of research participants. The swelling was 12.22±0.261 preoperatively at A, and after three days, it grew considerably to 13.53±0.242 (p = 0.00). In the surgical bur group, there was a noteworthy rise from 12.22±0.273 to 14.12±0.236 (p = 0.00). A substantial rise was seen in the laser group preop (A) to one week again, from 12.22±0.261 to 13.65±0.254 (p = 0.03), and in the bur group, from 12.33±0.273 to 12.97±0.256 (p = 0.00). A non-significant rise from preop A to 15 days was seen in Group I, where it increased from 12.22±0.261 to 13.53±0.242 with p = 0.34, and in Group II, from 10.34±0.273 to 12.32±0.266 with p = 0.134. There was a substantial rise for the bur group (p = 0.00) and a non-significant increase for the laser group (p = 0.16) from preop (B) to three days. Group I showed a non-significant rise (p = 0.59), but Group II (bur) showed a substantial increase (p = 0.01) following surgery (B). Table 2 shows that the laser group and bur group had a non-significant reduction from preop (B) to 15 days, with p = 0.16 and 0.54, respectively.

**Table 2:** Comparison of the swelling in the two groups of study subjects SD: standard deviation

Swelling	Group I (Er:YAG laser)		Group II (surgical bur)	
	Mean ± SD	p-value	Mean ± S. D	p-value
Preop (A)	12.22±0.261	0.00	12.22±0.273	0.00
3 days	13.53±0.242		14.12±0.236	
Preop (A)	12.22±0.261	0.03	12.33±0.273	0.00
1 week	13.65±0.254		12.97±0.256	
Preop (A)	12.22±0.261	0.34	10.34±0.273	0.134
15 days	13.53±0.242		12.32±0.266	
Preop (B)	10.33±0.2057	0.16	10.33±0.176	0.00
3 days	10.27±0.233		11.16±0.184	
Preop (B)	10.33±0.2057	0.59	10.33±0.176	0.01
1 week	10.02±0.173		10.66±0.157	
Preop (B)	10.33±0.2057	0.16	12.23±0.196	0.54
15 days	9.67±0.1476		10.33±0.154	

For mouth opening, the preoperative mouth opening was comparable in the two groups, with p = 0.87. On day 1 following extraction, in the laser group, the mean mouth opening was 18.22±0.49 mm and was significantly higher in the bur group with 22.47±0.89 mm with p = 0.000. After two days, mouth opening in the laser group was 23.93±0.54 mm, which was significantly lower compared to the bur group, where it was 28.28±0.45 mm with p = 0.000. Mouth opening in Group II was substantially greater than in Group I after three days and one week (p = 0.000). Table 3 indicates that, at the two-week mark, mouth opening was substantially greater in Group II (34.92±0.34 mm) than in Group I (32.52±0.54 mm), with p = 0.03. Additionally, according to the study's findings, there was no discernible difference between the two groups' socket healing (p < 0.05) based on a clinical evaluation of the extraction socket's healing. On the recall appointment, however, two patients from the laser group and four from the bur group had dry sockets. Two participants from Group II had paraesthesia when the surgical bur was used to cut bone, as shown in Table 3.

**Table 3:** Comparison of the trismus in the two groups of the study subjects SD: standard deviation

Mouth opening assessment	Mean ± SD	p-value
Preoperative		
Group I (Er: YAG laser)	31.83±0.61	0.87
Group II (surgical bur)	31.68±0.56	
1 day		
Group I (Er: YAG laser)	18.22±0.49	0.000
Group II (surgical bur)	22.47±0.89	
2 days		
Group I (Er: YAG laser)	23.93±0.54	0.000
Group II (surgical bur)	28.28±0.45	
3 days		
Group I (Er: YAG laser)	26.42±0.56	0.000
Group II (surgical bur)	30.52±0.39	
1 week		
Group I (Er: YAG laser)	30.87±0.45	0.000
Group II (surgical bur)	33.47±0.54	
2 weeks		
Group I (Er: YAG laser)	32.52±0.54	0.03
Group II (surgical bur)	34.92±0.34	

**Figure 1:**
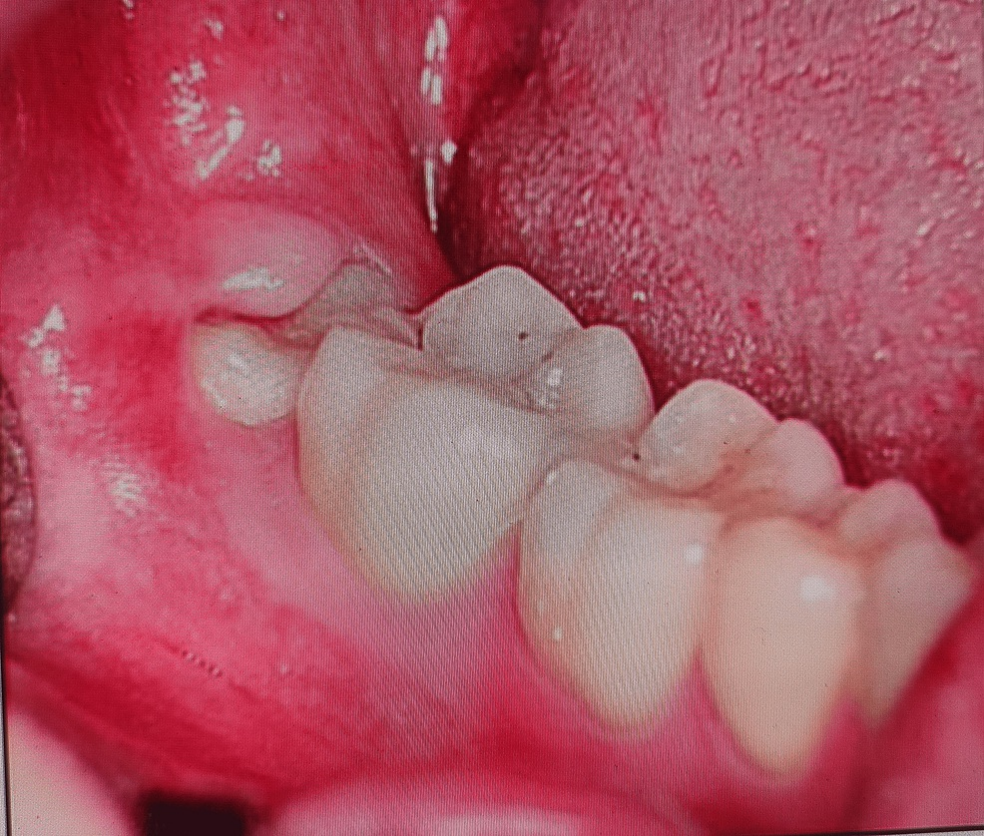
Group l (laser) preoperative view of an impacted mandibular third molar

**Figure 2:**
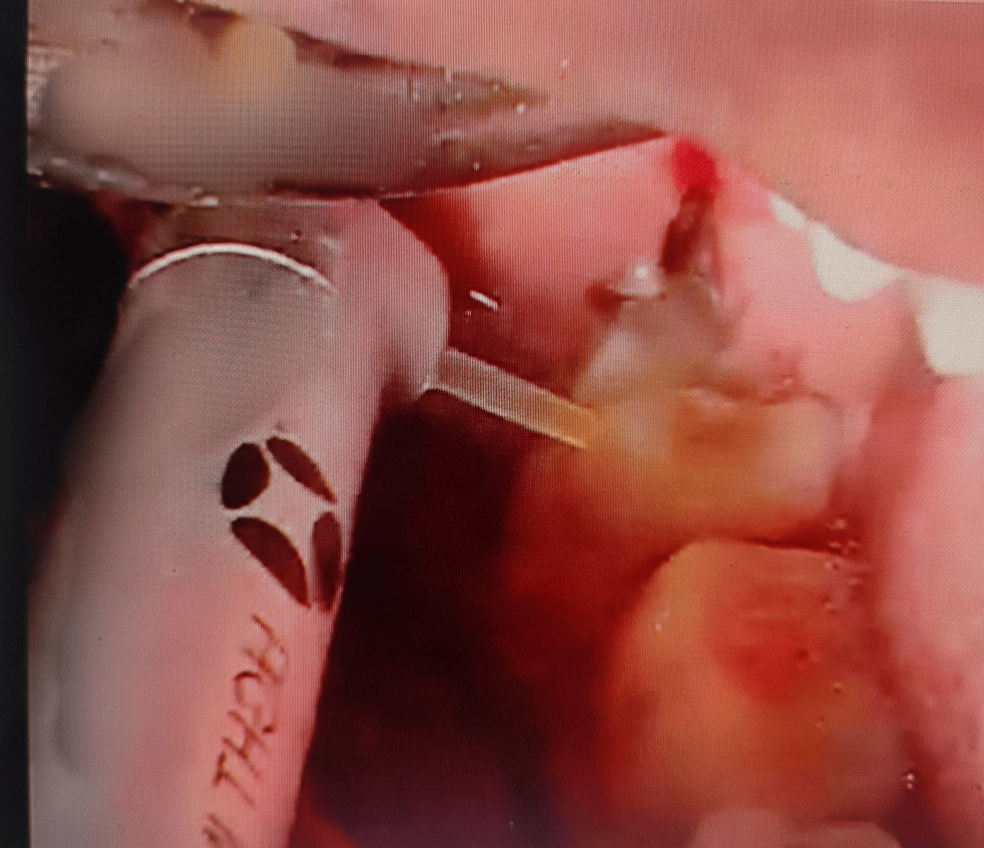
Group l intraoperative view of an impacted mandibular third molar by an Er:YAG laser

**Figure 3:**
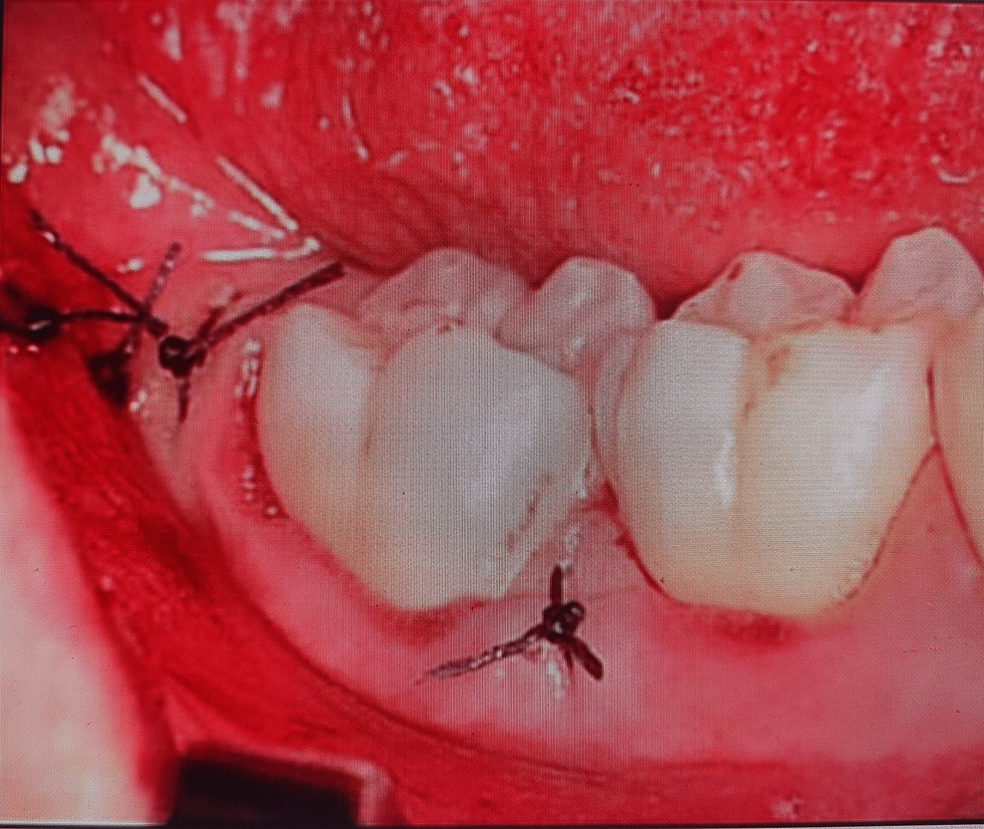
Group l postoperative view of the impacted mandibular third molar after laser use

Preoperative, intraoperative, and postoperative images in the laser group are presented in Figures 1-3, respectively.

**Figure 4:**
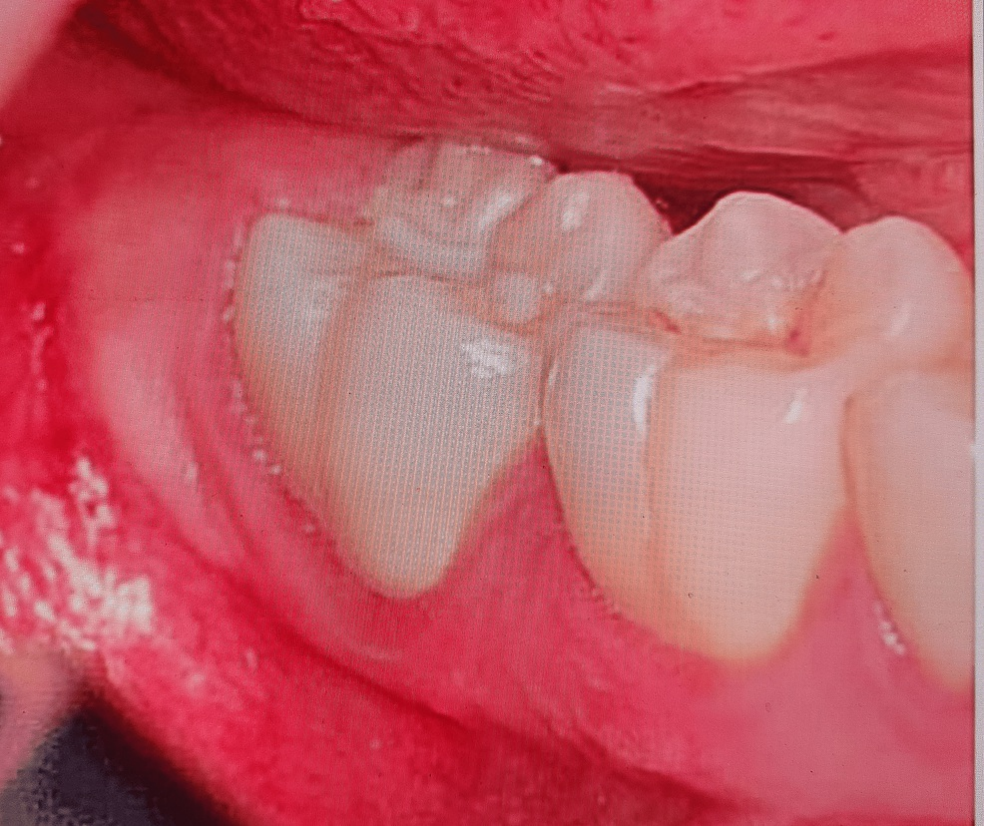
Group ll preoperative view of an impacted mandibular third molar using a surgical bur

**Figure 5:**
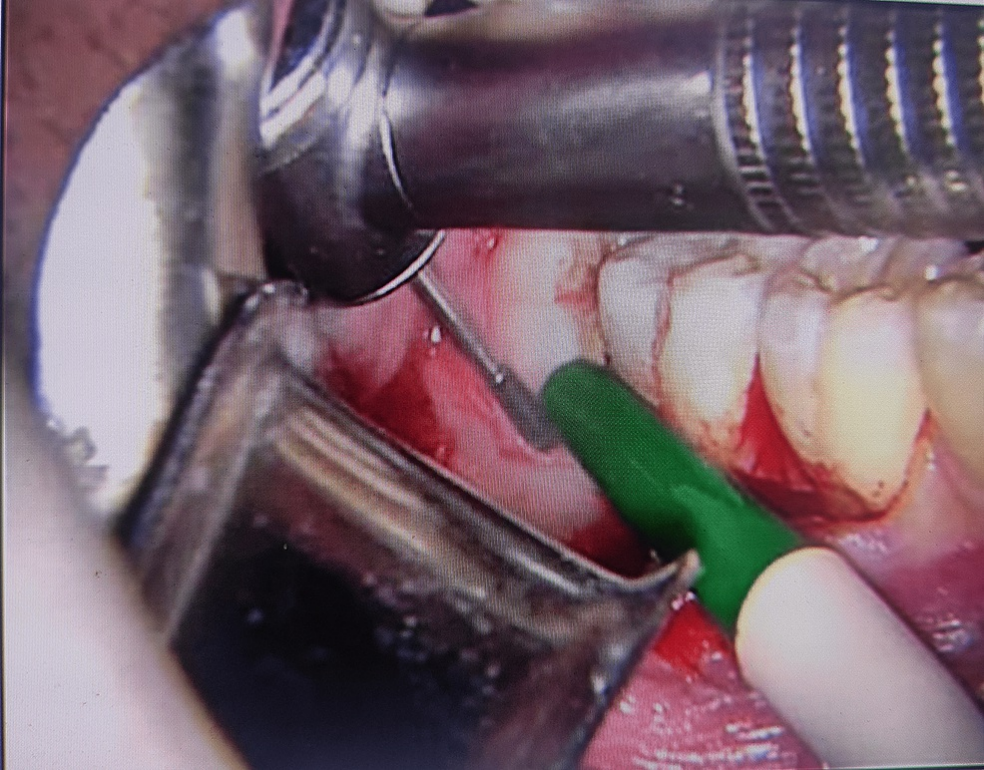
Group ll intraoperative view of the impacted mandibular third molar using a surgical bur

**Figure 6:**
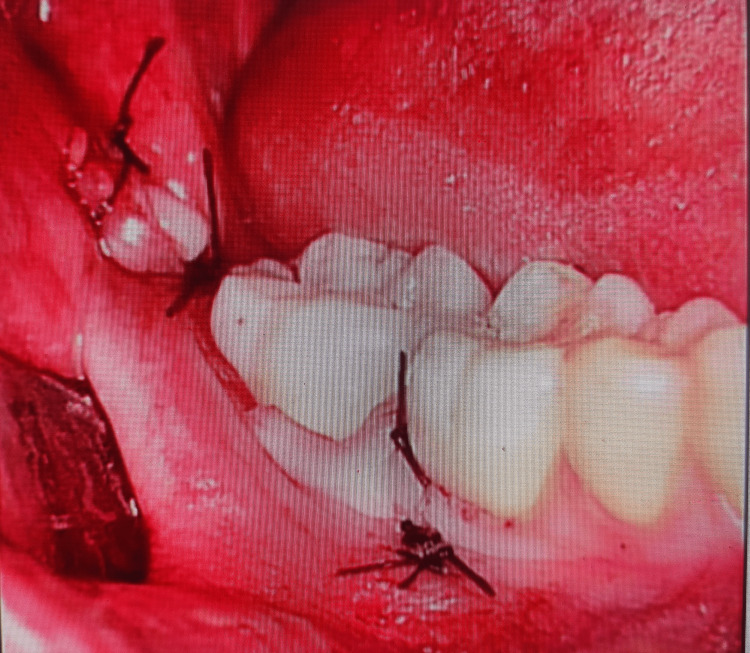
Group ll postoperative view of the impacted mandibular third molar using a surgical bur

The preoperative, intraoperative, and postoperative images with bur use (Group ll) are presented in Figures 4-6, respectively. The results show that postoperative bleeding and swelling are lesser in the laser group.

## Discussion

Eighty individuals, 40 of whom were male and 40 were female, participated in this study. Group I consisted of Er:YAG laser users, while Group II consisted of surgical bur users. The purpose of the two groups was to remove bones from the impacted teeth using an Er:YAG laser and a surgical bur, respectively. For the Group I and Group II research subjects, the mean age of the participants was 26.4±4.24 and 26.6±4.82 years, respectively. In Group I of the research, there were 55% (n = 22) men and 45% (n = 18) females; in Group II, there were 40% (n = 16) males and 60% (n = 24) females. The study sample consisted of 10% (n = 4) disto-angular, 5% (n = 2) horizontal, and 85% (n = 34) mesio-angular impaction participants. 

According to Passi et al. [1] in 2013 and Civak et al. [3] in 2021, pain, hemorrhage, and edema were among the clinical indicators that were reduced in the laser group compared to the bur group; however, the difference was not statistically significant. Postoperative swelling, however, revealed a notable distinction between the two groups. It took the laser group nearly twice as long to cut a bone using a bur. Trismus remained in the laser group for a longer duration. Clinical assessments of wound healing and sequelae revealed no discernible differences between the two groups.

Time taken was higher with a laser, which is seen in cases where the bone is thick. A bone has mineral salts that reduce vaporization causing a high operative time. The subject should be pre-informed about the prolonged time, as reported by Martins et al. [8] in 2011. A study by Papadaki et al. [9] in 2007 and Stübinger et al. [10] in 2008 reported a significant reduction in the operative time from 28 minutes to 5.33 minutes with increased energy per pulse with the most efficient setting being 2000 mJ/pulse. Nogueira et al. [11] in 2023 mentioned that even with longer surgical times, a significant reduction in operative time from 28 minutes to 5.33 minutes was obtained. He also mentioned that a piezo proved to be beneficial in reducing pain, swelling, and trismus following third molar extractions. These findings are also in coordination with our findings.

Abu‑Serriah et al. [12] in 2004 also mentioned that in the laser side, there was less edema (48 and 120 hours after surgery) and pain (24, 48, and 120 hours after surgery) (p < 0.05). In addition, 120 hours following surgery, laser-side edema and pain severity completely disappeared (p < 0.05).

Group I, which employed an Er:YAG laser, experienced wound healing in six days, whereas Group II (surgical bur) had wound healing in eight days. With p > 0.05, the surgical bur caused a non-significantly longer wound healing period. In Group I, 100% of the patients (n = 40) and, in Group II, 92.5% of the individuals (n = 37) experienced mild bleeding. No participants in the laser group and 7.5% (n = 3) of the surgical bur group had moderate bleeding. Neither group's subjects had severe bleeding. With p > 0.05, the difference in bleeding between the two groups was statistically not significant. These findings were in line with the earlier research conducted in 2021 by Nehme et al. [13], who found that between the baseline and the third day post-op, the "piezosurgery without dexamethasone" group showed the highest average (p < 0.0001). The "conventional rotatory without dexamethasone" group reported the highest mean (mean difference = 9.7, SD = 4.5, p < 0.0001).

According to a 2003 study by Takamori et al. [14], bleeding was less common with lasers because they have built-in hemostasis capabilities that alter protein functional activity in a way that is directly correlated with radiation dosage and frequency. According to the study's findings, 90% (n = 36) of the patients in Group I and 80% (n = 32) of the subjects in Group II reported having minor discomfort. A small portion (7.5%, n = 3) of the respondents in Group I and 5% (n = 2) of the subjects in Group II reported having moderate discomfort. Meanwhile, 2.5% (n = 1) of the respondents in Group I and none of the subjects in Group II reported having severe discomfort. The difference in the two groups was statistically non-significant, with p > 0.05.

These findings corroborated earlier research by Basheer et al. [15] in 2017, which reported that the rotary group experienced more severe pain until the fourth postoperative day, a difference that was statistically significant (p < 0.005). Up to the seventh postoperative day, mouth opening was noticeably better in the piezoelectric group when compared to the rotary bur group. Mozzati et al. [16] in 2011 found that pain was more severe following surgery. These findings can be linked to degeneration of nerve endings and disruption of nerve terminals in the bone.

The study's findings also revealed that the prevalence of edema was 12.22±0.261 prior to surgery; however, after three days, it grew dramatically to 13.53±0.242 (p = 0.00). In the surgical bur group, there was a noteworthy rise from 12.22±0.273 to 14.12±0.236 (p = 0.00). A substantial rise was seen in the laser group preop A to one week again, from 12.22±0.261 to 13.65±0.254 (p = 0.03) and from 12.33±0.273 to 12.97±0.256 (p = 0.00) in the bur group.

The study results also showed that preoperatively at A, the swelling was 12.22±0.261, which significantly increased to 13.53±0.242 after three days with p = 0.00. A similar significant increase was seen in the surgical bur group from 12.22±0.273 to 14.12±0.236, with p = 0.00. In the laser group preop A to one week again, a significant increase was seen from 12.22±0.261 to 13.65±0.254 with p = 0.03, and for the bur group, an increase was seen from 12.33±0.273 to 12.97±0.256 with p = 0.00. From preop A to 15 days, a non-significant increase was seen from 12.22±0.261 to 13.53±0.242 with p = 0.34 in Group I and from 10.34±0.273 to 12.32±0.266 in Group II with p = 0.134. From preop B to three days, a non-significant increase was seen with p = 0.16 for the laser group and a significant increase for the bur group with p = 0.00. From preop B to one week, a non-significant increase was seen in Group I with p = 0.59 and a significant increase in Group II (bur) with p = 0.01. A non-significant decrease was seen in the laser group and bur group from preop B to 15 days with p = 0.16 and 0.54, respectively. These results were in line with the previous studies of Takomari et al. [14] in 2003 and Coulthard et al. [17] in 2014 and Maiti et al. [18] in 2021, which revealed that more swelling is reported with the use of the surgical bur compared to the lasers.

For mouth opening, the preoperative mouth opening was comparable in the two groups with p = 0.87. On day 1 following extraction, in the laser group, the mean mouth opening was 18.22±0.49 mm, which was significantly higher in the bur group with 22.47±0.89 mm with p = 0.000. After two days, mouth opening in the laser group was 23.93±0.54 mm, which was significantly lower compared to the bur group where it was 28.28±0.45 mm with p = 0.000. After three days and one week, mouth opening was significantly higher in Group II compared to Group I with p = 0.000. After two weeks, mouth opening was significantly higher in Group II compared to Group I with 34.92±0.34 mm and 32.52±0.54 mm, respectively, with p = 0.03.

Similar to Coulthard et al. [17] in 2014 and Sales et al. [19] in 2022, reduced mouth opening was greater following laser treatment than with surgical bur. The extensive scope, seriousness of purpose, and bone loss have all been connected to these discoveries. The extended flap manipulation after the third molar removal also resulted in trismus.

The study's findings further demonstrated that, according to a clinical evaluation of the extraction socket's healing, there was no discernible difference between the two groups' socket healing (p < 0.05). On the recall appointment, two individuals in the laser group and four individuals in the bur group, however, had dry sockets. Two participants from Group II who had their bones sliced with a surgical bur reported experiencing paraesthesia. These results were in line with earlier research by Mozatti et al. [16] in 2011, Coulthard et al. [17] in 2014, and Maiti et al. [18] in 2021, in which the authors proposed that bur and laser might provide socket healing that was equivalent. Postoperative swelling, however, revealed a noteworthy distinction between the two groups. It took the laser group nearly twice as long to cut bone using a bur. Trismus remained in the laser group for a longer duration. Clinical assessments of wound healing and sequelae revealed no discernible differences between the two groups.

According to Sales et al. [19] in 2022, the findings of meta-analysis revealed that, in comparison to the drill group, the laser group experienced a significant decrease in edema (1.82 (95% CI = -3.06 to -0.57) cm) and complications (p = 0.0004), a slight decrease in pain after two days (p = 0.030), and no change in trismus (p = 0.200). In the drill group, the surgery and/or osteotomy took less time, which are consistent with our study.

Limitations

The study's limited sample size and the fact that only participants from a specific region were taken into account restrict the applicability of the findings to the larger Indian population. Because the research individuals were evaluated at a particular moment in time, long-term consequences were not taken into account.

## Conclusions

In two groups of 80 patients, the study compared the effectiveness of an Er:YAG laser for cutting bone and extracting the impacted mandibular third molar using a surgical bur. In Group I patients, swelling, bleeding, and discomfort were reduced when the laser was used instead of a surgical bur, but the difference was not statistically significant. 

The time needed for bone cutting was significantly more for laser compared to the surgical bur use where the time was nearly double. Concerning complications and wound healing, there was no discernible variation between the two groups. Trismus was present in Group I (laser) for a longer period of time.

Due to its less invasive nature, simpler procedure, and easier osteotomy than a traditional surgical bur, the study suggests that laser can be used for intra-oral bone cutting. Furthermore, the Er:YAG laser can be a better alternative to surgical burs in apprehensive subjects.
